# Dataset on modelling natural surfactant adsorption derived from non-edible seed oil (linseed oil) on sandstone reservoir rock

**DOI:** 10.1016/j.dib.2023.109578

**Published:** 2023-09-20

**Authors:** Kehinde Awelewa, Fred Ogunkunle, Oluwasanmi Olabode, Babalola Oni, Damilola Abraham, Samuel Adeleye, Samuel Ifeanyi

**Affiliations:** a Department of Petroleum Engineering, Covenant University, Canaan Land, Km 10 Idiroko Road, PMB 1023, Ota, Ogun State, Nigeria; b Department of Chemical Engineering, Covenant University, Canaan Land, Km 10 Idiroko Road, PMB 1023, Ota, Ogun State, Nigeria

**Keywords:** Surfactant adsorption, Chemical enhanced oil, Recovery, Interfacial tension

## Abstract

Surfactant flooding is adjudged one of the most promising chemicals enhanced oil recovery (cEOR) methods due to its high microscopic sweep efficiency. This surfactant shows high potential in mobilizing trapped residual oil (ganglia) through excellent lowering of the interfacial tension (IFT) between the crude oil-aqueous interface to ultra-low values while favorably altering the wettability (oil-wet to water-wet). Surfactant adsorption is a critical factor that determines how successful this cEOR method will be as well as the project economics. Surfactant retention due to adsorption caused majorly by electrostatic forces of attraction between hydrophilic head, and the positive and negative charges of the adsorbent solid surface leading to insufficiency of the remaining surfactant concentration in the injected slug to achieve the supposed ultralow IFT needed for mobilization. This article describes the experimental data on the adsorption of a natural surfactant derived from linseed oil and the results from its adsorption isotherm modelling. This anionic surfactant (LSO-derived) has a CMC value of 2500 ppm, average fractional removal of 0.60 under a range of concentrations (500, 1000, 2000, 4000, 8000, and 12000 ppm), with the adsorption kinetics revealing that adsorption density rises as a function of time with increasing adsorbate concentrations. Five different classical **adsorption isotherm models** were explored- in the form **three** (Redlich–Peterson or R-P**), two** (Langmuir, Freundlich, Temkin), **one** (Linear-Henry) **parameters** models. Their characteristics adsorption parameters were calculated, with highest adsorption capacity value of 2.955mg/g obtained from the simulation using OriginPro 2021 Software. The analysis demonstrates that the R-P model provided the greatest fit as a hybrid model with the highest correlation coefficient value. The kinetic adsorption models Pseudo-First Order (PFO), Pseudo-Second Order (PSO), Pseudo-Nth Order (PNO), and Intra-Particle Diffusion (IPD), as well as their thermodynamic property model, were also examined in addition to static isotherm models. On average, using non-linear regression approach, PSO and PNO provided the best appropriate fit models under this hypothesis, with correlation values of the nth order ranging from 0.443 to 2.122 (excluding 5.847 the non-converged fit value). Prior to thermodynamic analysis, it was confirmed by the IPD with multi-linear graphical characteristics that intra-particle transport was not the only rate-limiting step in adsorption processes and proceeded spontaneously by the This model can be utilized to design a template for LSO surfactant-rock adsorption in chemical flooding schemes for EOR applications.

Specification Table

**Table:** 

Subject	Petroleum Engineering

Specific subject area	Reservoir Engineering (CEOR), Surfactant adsorption, and adsorption mechanism
Data Format	Raw, analyzed.
Type of data	Table, Figures, and Graphs
Data collection	*How data were acquired:* The surfactant adsorptions on sandstone surfaces were monitored via the batch experiment approach at laboratory conditions of (**25** °C and atmospheric pressure). Using an Omega CDS107 multi-purpose conductivity meter, the conductivity of the surfactant solutions was monitored as a function of days, from which the adsorption was estimated. *Description of data collection:* For adequate data collection representative, the Berea core sample was crushed mechanically, dried at 100°C for 48 hours, sieved for average grain size of 325.5µm-the adsorbent. The adsorption kinetics process was experimentally investigated with a probing tool at air-aqueous interface, at laboratory temperature (25°C) to monitor the uptake of solutions (500-12000 ppm variant surfactant concentrations in deionized neutral PH medium) in relation to the adsorbent surface as a function of time-24hrs interval. The adsorption parameters for all isotherm and thermodynamics models for process conditions were modelled using OriginPro 2021 Software.
Data source location	Department of Petroleum Engineering, Covenant University, Ota, Nigeria
Data Accessibility	1. Data is with article. 2. Repository name: Green Surfactant Adsorption. 3. https://data.mendeley.com/datasets/btp4xf5v3z/2 DOI:10.17632/btp4xf5v3z.2

## 1 Value of Data

• The data will introduce maximum surfactant concentration, Cᵢ, at initial condition before equilibration, surfactant concentration, C_e_, after equilibration with sandstone grains, bulk volume of solution, V, used, and mass of adsorbent, M, (crushed sandstone).
• The data will provide adsorption densities, ꟼ_e_, at various time intervals until equilibration point is reached which is the maximum adsorption point.
• Through the data analysis from the static models, kinetic models, thermodynamic models, correlation coefficients, characteristics adsorption parameters provide the best fitting model for the experimental adsorption.
• Interpreting and predicting adsorption behavior through these models at ambient laboratory conditions leads to effective design of adsorption processes, which can be developed into a more robust/scale up systems for optimal recovery efficiency.
• These data offer important insight for academic researchers and industrial professionals involved in chemical enhanced oil recovery processes.
• As phenomenon data, this gives an indication of how economically viable the project of surfactant injection in cEOR.

## 2 Objective

This study quantifies an anionic surfactant adsorption density on to sandstone surfaces via a batch experiment, and as well model the adsorption isotherms from the raw experimental data for effective and efficient designing of a chemical enhanced oil recovery. Adsorption experimental regression modelling, illuminate the mechanism of surfactant adsorption phenomena in the course of surfactant flooding while exploring established adsorption isotherms such as the static, kinetic, and thermodynamic models at given temperature and contact time. On validation, model with minimal adsorption value is leverage on to determine among other factors the economic feasibility of a projected or proposed cEOR design.

## 3 Data Description

The primary raw data (Table 1) from surfactant (LSO) adsorption through Batch laboratory experimental processes was carried out with varying concentrations of 500 -1200 ppm. The isotherm-kinetic modelled data (Table 2) from raw data collected were analyzed with parameters for isotherms presented in Table 3 while that of kinetic alongside the thermodynamic estimated value are presented in Table 4. The plots of adsorption density at varying limiting time steps for kinetic models using the raw data are presented in Figs. 8 and 9. (The corresponding files in csv format are also uploaded in Mendeley Repository, https://data.mendeley.com/datasets/btp4xf5v3z/2).

**Table 1:** Primary Data, LSO + DI.

Day	C_e_ Cond(µS) @ 500 ppm	ꟼ_e_ Adsorption Density (mg/g) @ 500 ppm	C_e_ Cond(µS) @ 1000 ppm	ꟼ_e_ Adsorption Density (mg/g) @ 1000 ppm	C_e_ Cond. (µS) @ 2000 ppm	ꟼ_e_ Adsorption Density (mg/g) @ 2000 ppm	C_e_ Cond. (µS) @ 4000 ppm	ꟼ_e_ Adsorption Density (mg/g) @ 4000 ppm	C_e_ Cond. (µS) @ 8000 ppm	ꟼ_e_ Adsorption Density (mg/g) @ 8000 ppm	C_e_ Cond. (µS) @ 12000 ppm	ꟼ_e_ Adsorption Density (mg/g) @ 12000 ppm
0	342	0	390	0	450	0	590	0	820	0	1250	0
1	320	0.121	350	0.22	380	0.385	502	0.484	700	0.66	1100	0.825
2	300	0.231	300	0.495	340	0.605	435	0.8525	610	1.155	1000	1.375
3	280	0.341	270	0.66	300	0.825	400	1.045	570	1.375	950	1.65
4	260	0.451	250	0.77	270	0.99	374	1.188	540	1.54	920	1.815
5	245	0.5335	225	0.9075	255	1.0725	355	1.2925	520	1.65	890	1.98
6	230	0.616	205	1.0175	232	1.199	330	1.43	490	1.815	870	2.09
7	180	0.891	180	1.155	210	1.32	300	1.595	470	1.925	850	2.2
8	152	1.045	150	1.32	185	1.4575	270	1.76	460	1.98	830	2.31
9	130	1.166	130	1.43	160	1.595	255	1.8425	450	2.035	810	2.42
10	121	1.2155	110	1.54	140	1.705	245	1.8975	440	2.09	790	2.53
11	110	1.276	105	1.5675	135	1.7325	235	1.9525	430	2.145	770	2.64
12	100.2	1.3299	103	1.5785	130	1.76	225	2.0075	420	2.2	750	2.75
13	98.8	1.3376	102.5	1.58125	129.5	1.76275	224.3	2.01135	419	2.2055	740	2.805
14	95.5	1.35575	102.2	1.5829	129.2	1.7644	224.1	2.01245	418.5	2.20825	739.5	2.80775
15	92.2	1.3739	102.1	1.58345	129	1.7655	223.5	2.01575	418	2.211	738	2.816

*Note*: At $t_{Day=0},C_{i}=C_{e}$

**Average Fractional Removal, R=** 0.617163, (from Eq. 1).

**Table 2:** Model extracted data.

Conc (ppm)	Cᵢ (mg/L)	C_e_ (mg/L)	ꟼ_e_ Adsorption Density (mg/g)	1/C_e_ (L/mg)	1/ꟼ_e_ (g/mg)	Log C_e_	Log ꟼ_e_	Ln C_e_	(KC_e_/ꟼ_e_ -1)	Ln (KC_e_/ꟼ_e_ -1)	Ln C_e_	Ln (C_e_/ꟼ_e_)
500	342	92.2	1.3739	0.010846	0.727855	1.964731	0.137955	4.52396	13.761127	2.62184771	4.52396013	4.206307
1000	390	102.1	1.58345	0.0097943	0.6315324	2.009026	0.199604	4.625953	13.182902	2.57892068	4.62595273	4.166347
2000	450	129	1.7655	0.0077519	0.5664118	2.11059	0.246868	4.859812	15.071844	2.71282835	4.8598124	4.291378
4000	590	223.5	2.01575	0.0044743	0.4960933	2.349278	0.304437	5.409411	23.388471	3.1522432	5.40941141	4.70842
8000	820	418	2.211	0.0023923	0.452284	2.621176	0.344589	6.035481	40.584478	3.70338567	6.03548143	5.242037
12000	1250	738	2.816	0.001355	0.3551136	2.868056	0.449633	6.603944	56.645767	4.03681726	6.60394382	5.568626

**Table 3:** Parameter analysis of LSO surfactant adsorption on rock interface using different isotherm models.

Isotherm Model	Correlations	Parameters			
		q_0_ (mg/g)	*K_L_* (L/mg)	*R_L_*	*R*^2^
Langmuir	1/*q_e_* = 32.7385/*C_e_* + 0.33843	2.955	0.0103	0.072 - 0.220	0.9325
		n	*K_F_* [(mg/g)/(mg/L)^n^]	*R*^2^	
Freundlich	$AptCommandA7FC{;}_{e}=0.3905{\left(C_{e}\right)}^{0.2969}$	3.3687	0.3905	0.9340	
		*B_T_* (J/mol)	*K_T_* (L/mg)	*R*^2^	
Temkin	$AptCommandA7FC{;}_{e}=0.6016\left(lnC_{e}\right)-1.2533$	0.6016	0.1245	0.9450	
		C (mg/L)	*K_H_* (L/g)	*R*^2^	
Henry	$AptCommandA7FC{;}_{e}=0.0020\left(C_{e}\right)+1.4051$	1.4051	0.0020	0.9166	
		α (L/mg)^β^	*K_RP_* (L/g)	β	*R*^2^
Redlich-Peterson	$ln\left(0.21996\frac{C_{e}}{AptCommandA7FC{;}_{e}}-1\right)=0.7316ln\left(C_{e}\right)-0.7745$	0.4609	0.2200	0.7316	0.9879

**Table 4:** Parameter analysis of LSO surfactant adsorption on rock interface using different adsorption kinetic and thermodynamic models.

	500 ppm	1000 ppm	2000 ppm	4000 ppm	8000 ppm	12000 ppm
Pseudo-first order model						
$K_{1}\;{\left(min\right)}^{-1}$	0.0573	0.1407	0.1721	0.2100	0.3135	0.2681
***q_e_***(mg/g)	2.5721	1.8997	1.9725	2.1354	2.2024	2.7777
***R*^2^**	0.9698	0.9884	0.9911	0.9903	0.9920	0.9761
Pseudo-second order model						
$K_{2}\;$(g/mg min)	0.0072	0.0385	0.0533	0.0697	0.1298	0.0829
$AptCommandA7FC{;}_{e}$(mg/g)	4.5311	2.7601	2.7101	2.7984	2.6821	3.4483
***R*^2^**	0.9684	0.9848	0.9902	0.9923	0.9980	0.9918
Pseudo-nth order model						
$K_{n}\;\left(min^{-1}\right){\left(mgg^{-1}\right)}^{1-n}$	0.1413	0.1795	0.1818	0.0584	0.1446	*2.02214E-5*
n	0.4425	0.5348	0.9228	2.1220	1.9098	*5.84687*
$AptCommandA7FC{;}_{e}$(mg/g)	1.3488	1.6220	1.9244	2.8872	2.6320	*6.6290*
***R*^2^**	0.9635	0.9931	0.9905	0.9917	0.9978	*0.9962*
Intra-particle diffusion model						
$K_{diff}$(mg/g. min ^0.5^)	0.4395	0.4799	0.5092	0.5590	0.56838	0.7213
C(mg/g)	-0.2813	-0.1258	-0.0416	0.0478	0.2621	0.2389
***R*^2^**	0.9268	0.9663	0.9780	0.9768	0.9408	0.9743
Gibbs Free Energy	-11.342kJ/mol					

**Fractional (adsorption) removal,**

(1) $$\begin{array}{c} R\;=\;\frac{\left(C_{i}-C_{f}\right)}{C_{i}} \end{array}$$

where $C_{i}$ and $C_{f}$ initial maximum and final concentrations in mg/L, R is the Fractional Removal.

(2) $$ꟼₑ\;=\;\frac{V\left(C_{i}-C_{e}\right)}{W}$$

where $C_{i}$ and $C_{e}$ both in mg/L or equivalent ppm are initial and equilibrium concentrations respectively. V(L) is the volume of surfactant solution, W(g) is the mass of the adsorbent-crushed sandstone grains, ꟼₑ(mg/g), the adsorption density.

**Langmuir isotherm model,**

It is an empirical model based on the following assumptions (a) monolayer adsorption, (b) homogeneous sites, (c) constant adsorption energy, and (d) no lateral interaction between the adsorbed molecules.

(3a) $$ꟼₑ\;=\;\frac{{ꟼ}_{o}K_{L}C_{e}}{1+K_{L}C_{e}}$$

In linear form,

(3b) $$\frac{1}{ꟼₑ}=\frac{1}{{ꟼ}_{o}K_{L}C_{e}}+\frac{1}{{ꟼ}_{o}}$$

(3c) $$\text{And}\;R_{L}=\frac{1}{1+C_{i}K_{L}}$$

where, $K_{L}$ is the Langmuir constant (L$mg^{-1}$), $R_{L}$ is the equilibrium parameter (dimensionless)

Four possible values of RL:

a. favorable (0 < R_L_< 1),
b. unfavorable (R_L_ > 1),
c. linear (R_L_ = 1),
d. Irreversible (R_L_ = 0).

**Freundlich isotherm model,**

Freundlich isotherm hypothesis: This assumes multilayers adsorption on heterogeneous sites with non-uniform distribution of adsorption heat and affinities over the heterogeneous surface.

(4a) $$ꟼₑ=K_{F}{C_{e}}^{\frac{1}{n}}$$

The various values of 1/n:

a. favorable adsorption (0 < 1/n < 1)
b. unfavorable adsorption (1/n > 1)
c. linear adsorption (1/n = 1)

In linear form,

(4b) $$\text{log}ꟼₑ\;=\;\frac{1}{n}\text{log}C_{e}+\text{log}K_{F}$$

where $K_{F}$ is Freundlich adsorption capacity, and $\frac{1}{n}$ is surface heterogeneity factor (or adsorption intensity).

**Temkin isotherm model,**

This model assumes multilayer adsorption under adsorbate-adsorbate interactions processes, and that the surfactant heat of adsorption decreases linearly (not logarithmically) as the solid surface area increases. It is valid for intermediate ion concentrations range.

(5a) $$ꟼₑ=\text{BLn}K_{T}C_{e}$$

In linear form,

(5b) $$ꟼₑ=\;\text{BLn}C_{e}+\text{BLn}K_{T}$$

where $K_{T}$ (L/g) is Temkin isothermal constant, B (J mol^−1^) is the heat of adsorption.

**Linear-Henry's isotherm,**

The model assumption is based on monolayer adsorption at initially low adsorbate concentrations so that all adsorbate molecules are without interaction with the neighboring molecules.

(6) $$ꟼₑ=K_{H}C_{e}$$

$K_{H}\;\left(\text{mg}L^{-1}\right)$ is Henry equilibrium constant.

**Redlich–Peterson isotherm model**

The Redlich-Peterson (R-P) isotherm is a three-parameter empirical adsorption model that incorporates elements from both the Langmuir and Freundlich isotherms and amends the inaccuracies. This does not exhibit ideal monolayer adsorption behavior. Being a hybrid version, the Redlich−Peterson isotherm can be used in both homogeneous and heterogeneous systems.

β an exponent that ranges between 0 and 1.

When β = 1, R-P is reduced to the Langmuir equation

When β = 0, R-P condenses to the linear isotherm model

(7a) $$ꟼₑ=\;\frac{K_{\text{RP}\;}C_{e}}{1+{\alpha C}_{e}^{\beta }}$$

In linear form,

(7b) $$\text{Ln}\left(K_{\text{RP}\;}\frac{C_{e}}{ꟼₑ}-1\right)=\beta \text{Ln}C_{e}+\text{Ln}\alpha$$

$K_{RP\;}\left(Lg^{-1}\right)$ is Redlich-Peterson isothermal constant, $\alpha \;{\left(Lmg^{-1}\right)}^{\beta }$ is RP constant, and βthe heterogeneity exponential constant which is dimensionless.

**Kinetic adsorption models,**

­ **Pseudo-First Order Model,**

In deferential form,

(8a) $$\frac{\delta ꟼ{;}_{t}}{\delta t}=K_{1}{\left({ꟼ}_{e}-{ꟼ}_{t}\right)}^{1}$$

where $K_{1}$ (1/min) is the first order rate constant and $q_{t}$ is the adsorption capacity at time, t.

Integrating over boundary conditions ($q_{t}=0,\;when\;t=0\;and\;q_{t}=q_{t},\;when\;t=t$).

In linear form,

(8b) $$\text{Ln}\left({ꟼ}_{e}-{ꟼ}_{t}\right)=\text{Ln}ꟼ{;}_{e}-K_{1}t$$

In non-linear form,

(8c) $${ꟼ}_{t}={ꟼ}_{e}\left(1-e^{-K_{1}t}\right)$$

­ **Pseudo-Second Order Model**,

In differential form,

(9a) $$\frac{\delta ꟼ{;}_{t}}{\delta t}\;=K_{2}{\left({ꟼ}_{e}-{ꟼ}_{t}\right)}^{2}$$

where $K_{2}$(g/mg.min), is the second order rate constant.

Integrating with boundary conditions at $q_{t}=0,\;when\;t=0\;and\;q_{t}=q_{t},\;when\;t=t$.

In linear form,

(9b) $$\frac{t}{{ꟼ}_{t}}=\frac{1}{{K_{2}ꟼ}_{e}^{2}}+\frac{t}{{ꟼ}_{e}}$$

In non-linear form,

(9c) $${ꟼ}_{t}=\frac{K_{2}{ꟼ}_{e}^{2}t}{{1+K_{2}ꟼ}_{e}t}$$

­ **Pseudo-Nth Order Model**,

In differential form,

(10a) $$\frac{\delta ꟼ{;}_{t}}{\delta t}=K_{n}{\left({ꟼ}_{e}-{ꟼ}_{t}\right)}^{n}$$

where $K_{n},[$($min^{-1}){\left(mgg^{-1}\right)}^{1-n}]$ is the nth order rate.

After integration, the below is the non-linear form was obtained

(10b) $${ꟼ}_{t}={ꟼ}_{e}-{\left[\left(n-1\right)K_{n}t+{ꟼ}_{e}^{\left(1-n\right)}\right]}^{\frac{1}{1-n}}$$

­ **Intra-Particle Diffusion Model**,
  (11) $${ꟼ}_{t}=K_{\text{diff}}t^{0.5}+C$$

where $K_{diff}$ (mg/g$.min^{0.5})$, is the rate constant of IPD and C(mg/g) is the boundary layer or surface adsorption thickness.

**Thermodynamics Parameter**

­ **Gibbs Free Energy,**
  (12) $$\Delta G^{0}=\text{RTln}K_{L}$$

where R= 8.314J/mol. K, the universal gas constant, $K_{L}$ is the Langmuir constant, and ΔG is the Gibbs free energy(J/mol)

## 4 Experimental Design, Materials and Methods

### 4.1 Materials

In this article, methyl esters from non-edible seed oil (LSO), core samples (Berea Sandstone), conductivity meter (CDS107 Model), chemical reagents of analytical grade such as sodium hydrogen carbonate (99 %), methanol (99.8 %), sodium hydroxide (98 %), carbon tetrachloride (99.9 %), diethyl ether (99.5 %), chlorosulfonic acid (98 %), n-butanol (99.5 %), and sulfuric acid (98.5 %)—were used. These chemicals were bought from Sigma-Aldrich through Covenant University outsourcing outlet-. Deionize- distilled water- to prepare various solutions.

### 4.2 Methods and experimental design

**Fig. 1:**
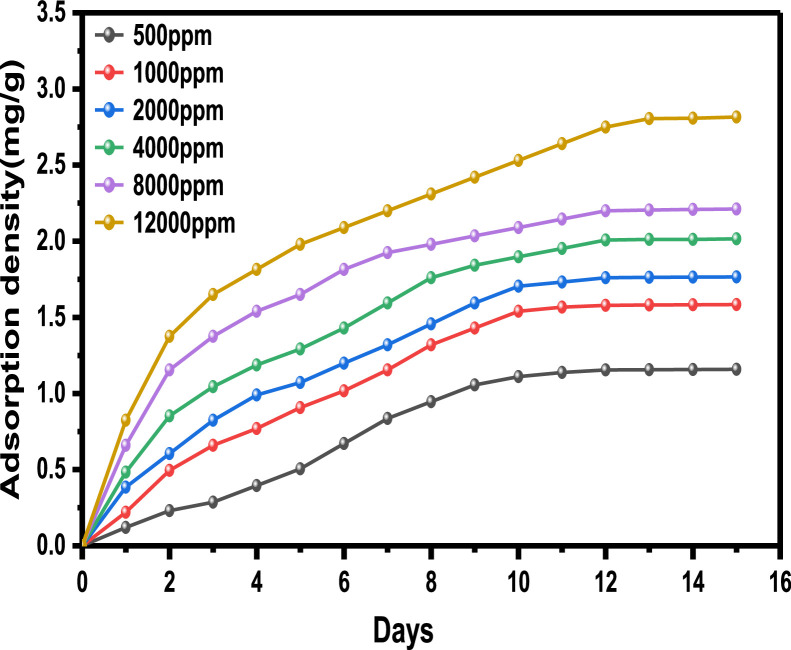
Adsorption density.

**Fig. 2:**
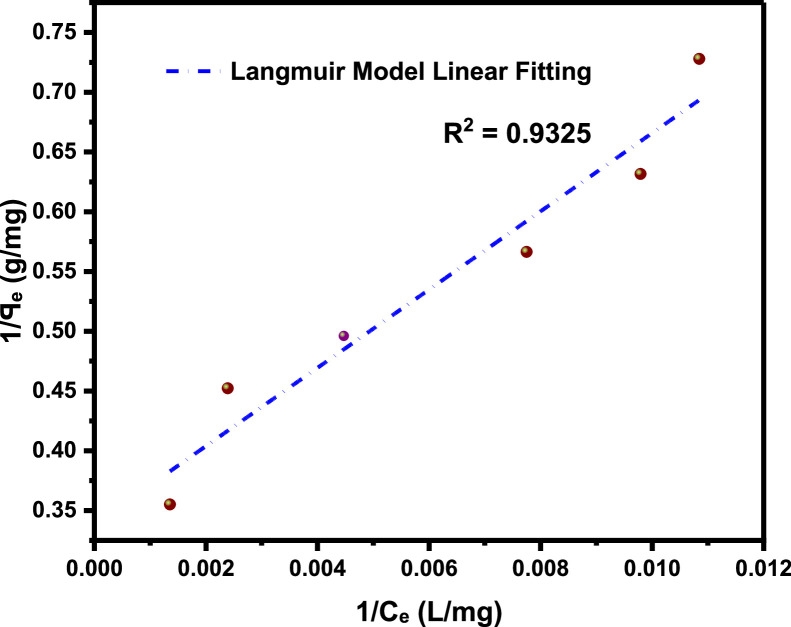
Langmuir model.

**Fig. 3:**
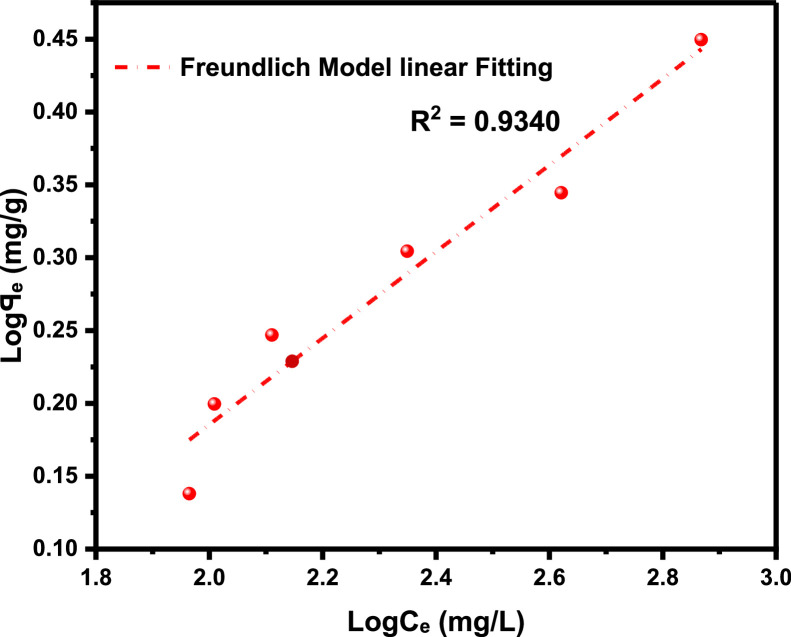
Freundlich model.

**Fig. 4:**
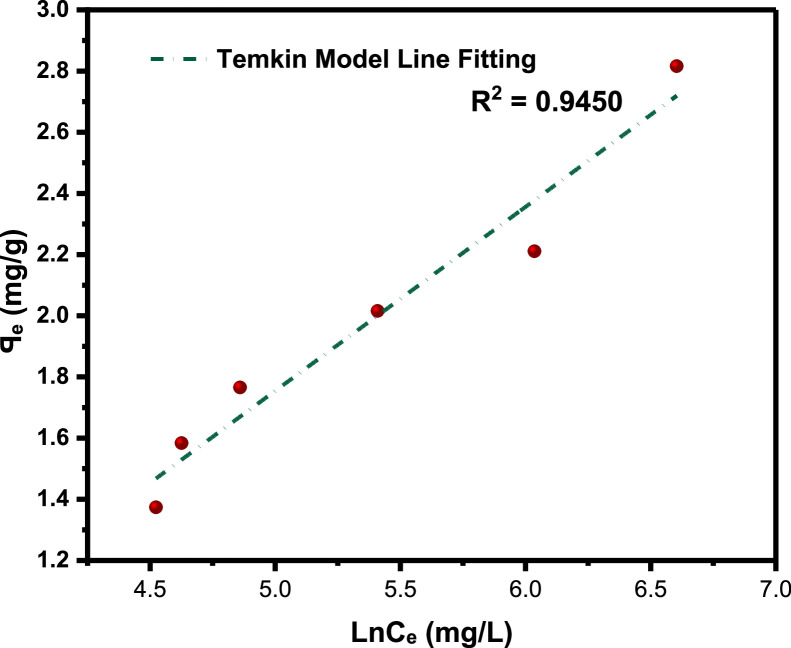
Temkin model.

**Fig. 5:**
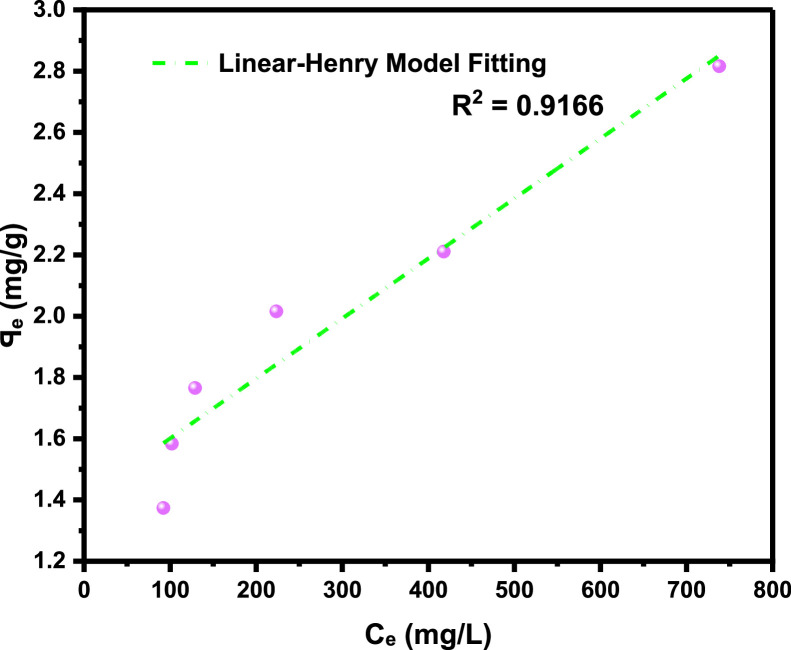
Henry model.

**Fig. 6:**
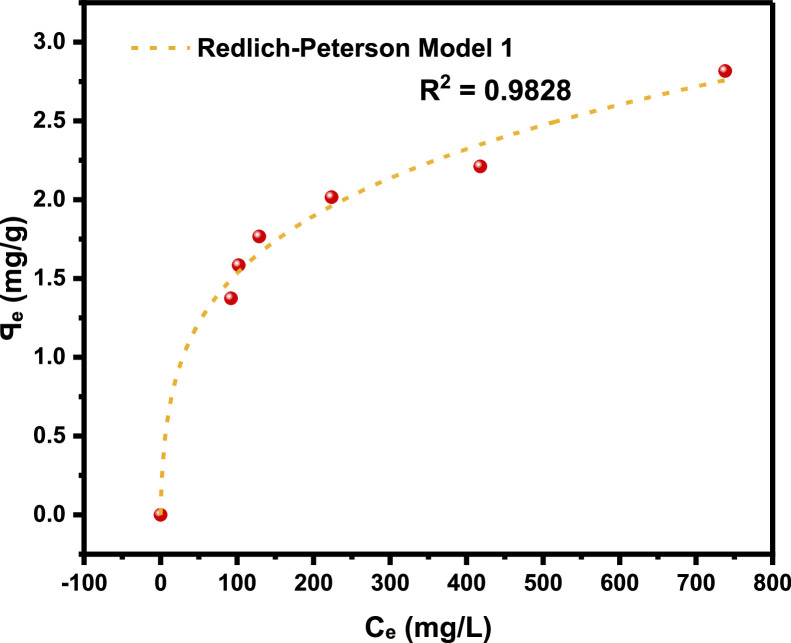
Non-linear Redlich-Peterson model.

**Fig. 7:**
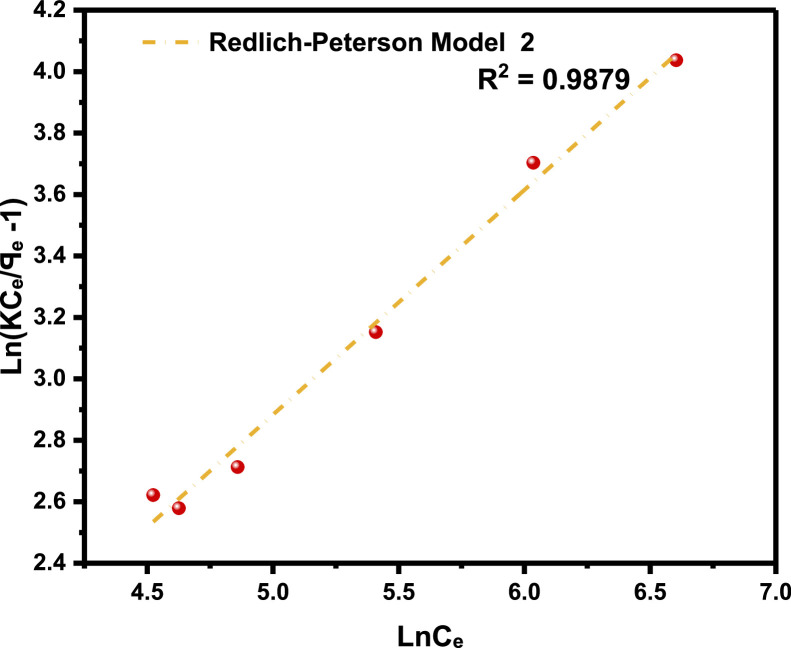
Redlich-Peterson model.

The anionic surfactant used in this work is derived from linseed oil, a triglyceride from the Linaceae family which also known flaxseed oil plant (*Linum usitatissimum*). It contains high dietary fibre with unusually large amount of α-linolenic acid(ALA), hence they are green and biodegradable [[1], [2], [3]]. This linseed derived surfactant are produced through a combination processes: transesterification and sulfonation as a means of improving the eventual surfactant stability in aqueous solution during chemical flooding applications [[4], [5], [6], [7]]. The adsorbent (from Berea sandstone core) is of broad distribution after being pulverized, dried, and sieved through different mesh sizes by a sieve-shaker with average gradation size of 325.5µm from 297µm to 354 µm sieve size. The conductivity meter (CDS107) a portable, high precision, multi-purpose microprocessor device that measures conductivity as well as other physical parameters including pH, ORP total dissolved solids, salt, and temperature of solutions by dipping the probe end of this instrument into the tested solution and digitally display the measurement reading once it has stabilized was used for conductivity measurements [8]. The effects of surfactant concentrations with respect to the fluids interfacial tension have been studied by [9] thus, for this experiment, different concentration: 500, 1000, 2000, 4000, 8000 and 12000 ppm of the synthesized surfactant were prepared via overhead stirring method. The ratio of surfactant solution to sandstone grains was 5:1 ratio, or 20 mL of the appropriate surfactant solution to 4 g of sandstone grains. Through Batch-experimental setup, adsorption densities were calculated from the conductivity measurements [10,11], to determine the LSO surfactant solution adsorption strength at varying concentrations (500-12000 ppm) as shown in Table 1. The successive difference between the initial surfactant concentration and the equilibrium concentration at referenced time with constant solution and adsorbent mass is used to calculate the surfactant mass-loss presented in this table in terms of adsorption density. Conductivity measurements were taken every 24 hours until equilibrium is attained which is equivalent to constant concentration, obtained by the difference in conductivity measured from the supernatant solution before and after equilibration—a point at which the residual concentration remained constant. Eq. 2 is used to calculate the magnitude of adsorption at subsequent equilibrium time steps (24 hours), and the entire data acquired is displayed in Table 1, the average **Fractional Removal, R** calculated **(from** Eq. 1**, and value given below** Table 1), and the adsorption density relationship as a function of time displayed in Fig. 1. Thereafter, five different established models which relate the amount of surfactant loss to the adsorbent at proportionally increasing concentrations were evaluated from the corresponding model **Equations 3 to 7** [[12], [13], [14], [15]]**.** For the plots, Table 2 is extracted from Table 1 to test for appropriate correlation which models adsorption behavior of the LSO surfactant under investigation. Parameters *q_e_,Ce*, 1/*q_e_*, 1/*C_e_*, log*C_e_*, etc., are computed to simulate the relationships for this behavior, with the results presented in Fig. 2, Fig. 3, Fig. 4, Fig. 5, Fig. 6, Fig. 7. For further insight, Kinetic adsorption models PFO, PSO, PNO (with fetched data from Table 2 and respective **Equations 8-10**), and IPD (from Table 1) were computed to evaluate the LSO-sandstone system as a rate limiting step [16]. Non-linear regression technique was used to evaluate the dependency of adsorption terms on concentration, do comparative analysis [17,18], to accurately predict the kinetic behavior from the experimental data at various initial concentrations of LSO, as well as a rate-limiting process in light of parameters related to the PFO, PSO, and PNO (Table 4.). As a complement rate limiting step, linear regression was used to assess and fit the IPD as mass transfer diffusion model (Eq. 11), which explains the LSO adsorption pattern on the sandstone as a function of time. This pattern is indicative of the rate at which the adsorbate and the adsorbent diffuse towards one another. To determining the spontaneity of the entire process [15]., the thermodynamic adsorption of the solution at 25°C(298.15K) was carried out to acquire thermodynamic parameter (Eq. 12) using Langmuir equilibrium constant, $K_{L},$ to evaluate change in ΔG (Table 4), a way to assess and analyze the impact of temperature [19]on LSO adsorption process.

**Fig. 8:**
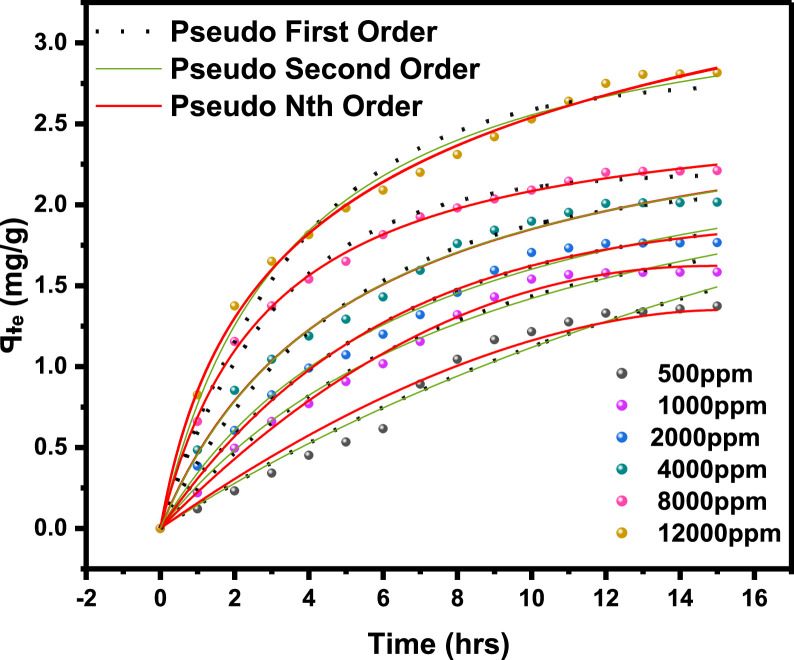
Comparative adsorptive kinetic models.

**Fig. 9:**
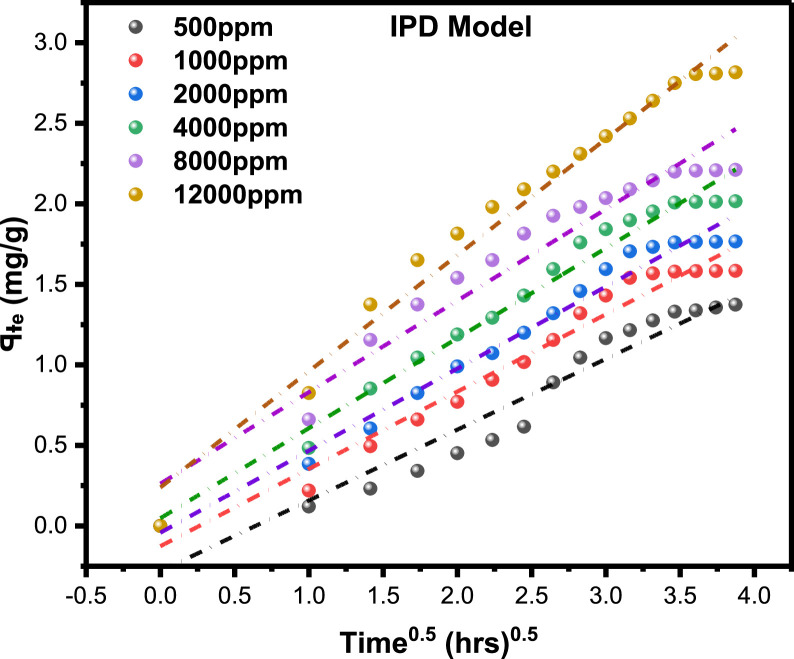
Infra-Particle Diffusion Model for LSO adsorption.

## Limitations

None.

## Ethics Statement

The data presented does not involve any experimentations on humans or animals.

## CRediT authorship contribution statement

**Kehinde Awelewa:** Conceptualization, Methodology. **Fred Ogunkunle:** Validation, Writing – review & editing, Supervision. **Oluwasanmi Olabode:** Writing – review & editing, Supervision. **Babalola Oni:** Writing – review & editing. **Damilola Abraham:** Methodology, Supervision. **Samuel Adeleye:** Writing – original draft. **Samuel Ifeanyi:** Writing – review & editing.

## Data Availability

Dataset on Modelling Natural Surfactant Adsorption Derived from Non-Edible Seed Oil (Linseed Oil) on Sandstone Reservoir Rock (Original data) (Green Surfactant Adsorption)
